# The Adverse Impact of Glaucoma on Psychological Function and Daily Physical Activity

**DOI:** 10.1155/2020/9606420

**Published:** 2020-04-21

**Authors:** Wenbin Huang, Kai Gao, Yaoming Liu, Mengyin Liang, Xiulan Zhang

**Affiliations:** ^1^State Key Laboratory of Ophthalmology, Zhongshan Ophthalmic Center, Sun Yat-sen University, Guangzhou, China; ^2^Hainan Eye Hospital and Key Laboratory of Ophthalmology, Zhongshan Ophthalmic Center, Sun Yat-sen University, Haikou, China

## Abstract

**Purpose:**

To evaluate the impact of glaucoma on vision-related quality of life and physical activity.

**Methods:**

This study included 50 glaucoma patients and 50 healthy control subjects. Sociodemographic and clinical data were collected from all subjects. A Chinese version of the NEI VFQ-25 was used to evaluate the quality of life. Objective physical activity was assessed by wearing an accelerometer for 7 consecutive days.

**Results:**

No significant difference was found in sociodemographic data between the two groups (all *p* < 0.05). Visual acuity and visual field scores were worse in the glaucoma group than in the control group (all *p* < 0.001). The VFQ-25 scores indicated significantly lower scores for ocular pain, social function, mental health, role difficulties, and color vision in the glaucoma group than in the normal group (all *p* < 0.05). The average daily step count was lower in the glaucoma group than in the normal group. High, moderate, and low average daily step counts in the glaucoma group were associated with early-, moderate-, and advanced-stage glaucoma, respectively, while the step count was significantly lower in the advanced-stage glaucoma group than in the control group (*p*=0.037). A positive relationship was found between the average daily step count and social function and mental health (both *p* < 0.05).

**Conclusions:**

We demonstrated an adverse impact of glaucoma on psychological function and daily physical activity. Social function and mental health showed declines in glaucoma patients, and physical activity was limited in patients with advanced-stage glaucoma.

## 1. Introduction

Glaucoma is a chronic disease that can cause severe visual impairment and even blindness. Previous studies have determined that glaucoma has a significant negative impact on the psychological, social, and emotional functioning and can leave affected persons with anxiety, poor self-image, poor psychological well-being, and reduced confidence in health care [1]. Modest correlations have been found between visual field losses and vision-specific dependency, role difficulties, social and emotional well-being, and mental health based on patient scores on the National Eye Institute Vision-Function Questionnaire (NEI VFQ-25) [2, 3].

In the past, in their diagnosis and treatment of patients with glaucoma, ophthalmologists have often focused on clinical indicators, such as intraocular pressure (IOP), visual field (VF), visual acuity (VA), and adverse reactions to antiglaucoma drugs, but they have paid little attention to their patients' quality of life and mental health. Physical activity is an important part of the human lifestyle for maintaining and improving quality of life and mental health, and several studies have found positive effects of physical activity on the quality of life of patients with chronic diseases and advanced cancer. For example, a physical exercise intervention study on cancer survivors conducted by Valenti et al. [4] found that jogging, brisk walking, cycling, and other moderate physical activities can reduce the body pain of survivors, help relieve psychological stress, enhance physical, psychological, and emotional function, and significantly improve the overall quality of life. Lowe et al. also found similar improvements in quality of life with appropriate physical activity [5]. However, at present, there are few studies have researched on the relationship between physical activity and improved quality of life and mental health in patients with eye diseases such as glaucoma.

The present study is to concurrently study the quality of life and mental health by using the NEI VFQ-25 score to evaluate the effects of physical activity, determined with an accelerometer, in patients with glaucoma. Understanding the effects of physical activity limitations imposed by glaucoma may be important, because targeted strategies aimed at assisting glaucoma patients with their daily physical activities could potentially improve both their mental health and their quality of life.

## 2. Methods

### 2.1. Statement of Ethics

This prospective study was carried out at the Clinical Research Centre of Zhongshan Ophthalmic Centre, Sun Yat-Sen University, Guangzhou, China, between June 2016 and November 2016. The study was approved by the Ethical Review Committee of the Zhongshan Ophthalmic Centre (2015MEKY094). All participants received a detailed explanation about the study and signed an informed consent form, in accordance with the principles embodied in the Declaration of Helsinki. All subjects were from the Chinese Han population.

### 2.2. Subjects and Enrolment Criteria

The subjects that met the following conditions were enrolled in the study: (1) retired Han Chinese residents of Guangzhou aged 55–75 years, male or female; (2) patients with glaucoma but without limb disability, who could walk outside and have no contraindications for physical activity; and (3) the best-corrected visual acuity greater than 20/40 in one of the eyes. A control group of healthy persons without glaucoma was also enrolled.

Patients diagnosed with primary angle-closure glaucoma (PACG) or primary open-angle glaucoma (POAG) were consecutively recruited from the Glaucoma Department, Zhongshan Ophthalmic Centre. The following diagnostic criteria of the International Society of Geographical and Epidemiological Ophthalmology (ISGEO) classification system [6] were used for PACG diagnosis: greater than 180° iris trabecular meshwork contact, accompanied by intraocular pressure ≥22 mmHg (measured by Goldmann applanation tonometer) and/or with surrounding adhesion of the anterior chamber, no adhesion of the anterior chamber caused by secondary causes, accompanied by glaucomatous optic nerve injury (cup/disc [C/D] ratio > 0.7 and/or C/D asymmetry > 0.2 and/or focal notching), and a corresponding visual field defect test. The diagnostic criteria for POAG were as follows: no adhesion of the anterior chamber, accompanied by intraocular pressure ≥22 mmHg, accompanied by glaucomatous optic nerve injury, and corresponding visual field defect. Normal subjects were included who had no prior history of eye disease other than cataract, no glaucomatous optic neuropathy, and no history of IOP exceeding 21 mmHg.

Exclusion criteria for participants enrolled in this study included the following: (1) a combination of glaucoma with other diseases of the eye, including corneal abnormalities or corneal infection, iris corneal endothelial syndrome, anterior segment hypoplasia, high myopia (>6.0*D*), uveitis, ocular tumor, ocular trauma, and fundus lesions such as central retinal vein occlusion, central retinal artery occlusion, and retinal detachment; (2) patients who were participating in other clinical trials; and (3) patients residing in a residential or nursing home or who were identified with systemic diseases or physical disabilities that were not suitable for performing daily living activities.

### 2.3. Baseline Assessment

Before ocular examination, trained interviewers administered a structured questionnaire, which included detailed demographic information (age, gender, educational level, marriage, and occupation), history of systemic disorders and ocular diseases, and lifestyle. All subjects underwent an ophthalmic evaluation, which included VA measurement, slit-lamp biomicroscopy examination, gonioscopy, IOP measurement (Goldmann applanation tonometry), fundus examination, and a VF test (the SITA standard algorithm with a 24-2 test pattern; Humphrey Visual Field Analyzer II, Carl Zeiss Meditec, Dublin, California, USA).

### 2.4. Visual Function Questionnaire (VFQ-25)

A Chinese version of the NEI VFQ-25, which was used in previous research [7, 8] and suggested as a reliable and valid tool for assessing the visual functions of Chinese patients with eye diseases, was administered to all the enrolled subjects. All subjects were requested to fill in the questionnaire on their own. A trained researcher explained the questionnaire to the participants and provided assistance when required. The completed questionnaires were reviewed by the researcher to ensure no data were missing. Each item of the NEI VFQ-25 in the questionnaire was assigned to one of the 12 subscales: general health, general vision, ocular pain, near activities, distance activities, social functioning, mental health, role difficulties, dependency, driving, color vision, and peripheral vision. Answers to each question on the VFQ-25 were converted to a 100-point scale (where 100 represents the best possible score or the minimal subjective impairment and 0 represents the worst or the maximal subjective impairment). The guidelines published by the National Eye Institute (NEI) were followed when calculating the above scale conversions and subscale scores.

### 2.5. Objective Physical Activity Assessment

A research assistant explained to each subject how to measure their usual physical activity levels for 7 days by wearing an accelerometer (Actigraph GT3X+) on a belt over the hip. The participants were asked to continue wearing the accelerometer to record physical activity (in 5 s epochs) all day for 7 consecutive days, except for bathing and swimming. Participants were requested to record the time and reason for not wearing the accelerometer per day. Accelerometers and diaries were returned to the practice. If the participants wore the accelerometers less than 10% of the time throughout the day, they were asked to repeat wearing the accelerometer.

ActiGraph data were analyzed using an Actilife software (v6.6.0) set to ignore runs of ≥60 minutes of zero counts [9, 10]. The analysis summary variables were as follows: daily step counts, accelerometer wear time, percentage of moderate- to vigorous-intensity physical activity (MVPA), sleep quality, and metabolic rate.

### 2.6. Statistical Analysis

The data were processed and analyzed statistically using STATA software, version 14.0 (STATA Corp., College Station, TX). Demographic data, as well as clinical measurements, were tabulated for all participants and by the two groups. The baseline clinical characteristics of the study subjects were analyzed by the *t*-test and chi-square test. The significance of differences between the glaucoma and normal groups was determined using the chi-square test for categorical variables, the t-test for normally distributed variables, and the Mann–Whitney *U* test for continuous variables that were not normally distributed. Accelerometer data among the normal group and in patients with different stages of glaucoma were analyzed using analysis of variance (ANOVA) and post hoc LSD tests. Regression analysis was performed to investigate the correlations between the daily step count and clinical variables. All tests were considered statistically significant at *p* < 0.05.

## 3. Results

### 3.1. Baseline Characteristics

In total, 50 glaucoma patients and 50 normal subjects were recruited in this study. Their demographic and clinical data are summarized in Table 1. No significant difference was found between the two groups in terms of age, gender, marital status, education level, status of smoking and drinking, chronic diseases, insurance, season of baseline measure, body mass index, and heart rate (all *p* > 0.05). As would be expected, IOP was significantly higher in the glaucoma group, while VA and VF were worse in the glaucoma group than in the control group (all *p* < 0.001).

### 3.2. VFQ-25 Scores

**Figure 1:**
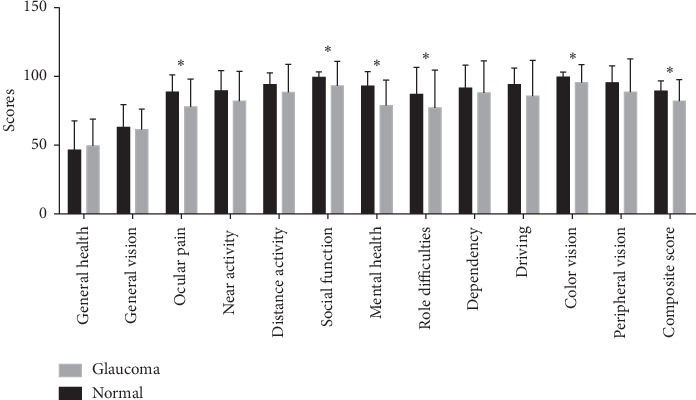
The mean scores for each subscale and composite score of the NEI VFQ-25 in patients with glaucoma and in healthy control subjects. The scores for ocular pain, social function, mental health, role difficulties, color vision, and composite score were significantly lower in the glaucoma group than in the normal group (^*∗*^*p* < 0.05).

The mean scores of each VFQ-25 subscale are shown in Figure 1, ranging from 49.48 for general health to 95.4 for color vision in the glaucoma group and ranging from 46.35 for general health to 99.47 for color vision in the normal group. The mean composite score was significantly lower in the glaucoma group than in the normal group (82.07 vs. 89.21, *p*=0.005). The analysis on each subscale score between the two groups revealed no significant differences in the scores for general health, general vision, near activity, distance activity, dependency, driving, and peripheral vision, whereas the scores for ocular pain,  social function, mental health, role difficulties, and color vision were significantly lower in the glaucoma group than in the normal group (all *p* < 0.05).

### 3.3. Objective Physical Activity Data

**Figure 2:**
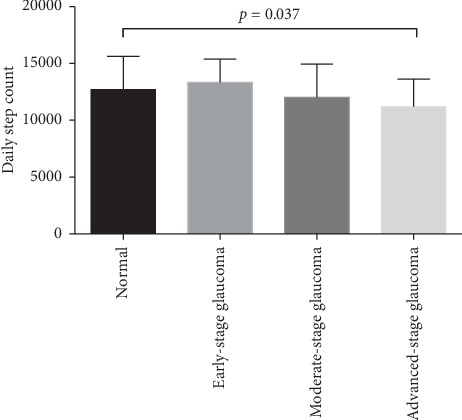
The average daily step count between patients with early-, moderate-, and advanced-stage glaucoma and healthy control subjects. The average daily step count was lower in the advanced-stage glaucoma group than in the control group (*p*=0.037).

The accelerometry data are shown in Tables 2 and 3. The average daily step count was lower in the glaucoma group than in the normal group, but the difference was not statistically significant (11682.22 vs. 12703.77, *p*=0.102). No significant difference was found in terms of the percentage of MVPA, sleep quality, and the metabolic rate. However, the subgroup analysis revealed a significantly lower average daily step count in the advanced-stage glaucoma group than in the control group (*p*=0.037) (Figure 2).

Linear regression analysis was used for further study of the relationship between the daily step count and clinical variables. The results are shown in Table 4. In the glaucoma group, the average daily step count was correlated with VA (both in the better eye and worse eye), VF (both in the better eye and worse eye), social function, and mental health (all *p* < 0.05), while in the normal group, the average daily step count was correlated with VA (in the better eye) and mental health (both *p* < 0.05).

## 4. Discussion

The quality of life of patients with glaucoma was first examined 20 years ago, and subsequent research has focused on the impact of glaucoma on patients' lives. Some population-based studies [11, 12] and multicenter clinical trials [13] have evaluated self-reported disability due to glaucoma. Recent work has assessed which activities are the most important for glaucoma patients by asking them to make choices in hypothetical situations in which they would expect to have different difficulties in performing different tasks.

In the present study, we found that the NEI VFQ-25 scores for ocular pain, social function, mental health, role difficulties, and color vision were significantly lower in the glaucoma group than in the normal group. These results were similar to those presented previously by Parrish et al. [2] and Gutierrez et al. [3], who found that vision-specific dependency, role difficulties, social and emotional well-being, and mental health were adversely affected in patients with glaucoma. Glaucoma can affect patients' quality of life and mental health from the following aspects [1, 14]: fear of social activities due to impaired visual function (poor peripheral vision and decreased VA), inconvenience caused by chronic use of eye drops, the psychological burden of a glaucoma diagnosis, adverse reactions to glaucoma treatment, and the increasing economic burden of a chronic disease such as glaucoma.

Difficulties with the extremes of vision are the most common complaints in glaucoma. Individuals with glaucoma have self-reported difficulty in living on their own due to difficulties in reading, walking, and driving [15]. Bilateral glaucoma is also associated with decreased driving ability, colliding with objects, slow walking, and falls, which adversely affect social activities and mental health. Walking and balancing are important for a healthy life, so when walking becomes difficult or is accompanied by fear of falls, patients may limit their physical activity [16] and thereby worsen their quality of life [17]. This, in turn, can lead to increased morbidity and higher mortality [18].

Most of the previous studies that have investigated physical activity in glaucoma patients have used questionnaire surveys. A questionnaire survey of glaucoma subjects showed that the glaucoma group mentioned walking difficulties as the most common complaints after visual impairment, with 49% describing difficult steps, 42% describing difficulty in shopping, and 36% describing difficulty in crossing the road [19]. Another questionnaire study found that VF defects led to bumping into objects and having difficulty with stairs [20].

In the present study, we used an accelerometer to measure the physical activity of patients with glaucoma to obtain more objective data than can be obtained with questionnaires alone. We found that the average daily step count was lower in the glaucoma group than in the normal group and that the average daily step count in the glaucoma group was inversely correlated with the stage of glaucoma; overall, patients with advanced-stage glaucoma were less active. This difference was statistically significant when patients with advanced-stage glaucoma were compared with healthy control subjects. The average daily step count in the glaucoma group was also correlated with worsened VA, VF loss, social function, and mental health. We hypothesize that visual impairment in patients with glaucoma has adverse effects on their mental health and their ability to function socially which, in turn, limits their physical activity. To the best of our knowledge, this is the first study to research the relationship between objective data of physical activity and the quality of life and mental health in patients with glaucoma. However, the causal relationship between these observations requires further research.

Physical activity is an important determinant of health and well-being in older people. An increase in physical activity levels can have modest but appreciable effects on the risks for important chronic diseases [21, 22]. A recent study showed that increased physical activity is associated with slower VF loss in patients with glaucoma [23]. Therefore, a prospective clinical study to establish suitable physical activities according to the physical and psychological abilities of patients with glaucoma could be meaningful. This type of study could also determine whether physical activity might enhance the social and psychological function and improve the quality of life of these patients.

Several limitations should be mentioned in our study. One limitation was that only retired individuals between the ages of 55 and 75 years were studied, and our findings may not generalize to patients in younger or older age ranges. Then, the physical activity may also differ considerably in regions with different weathers, different built environments, or different views about activity and exercise, which may have greater or lesser effects on daily physical activity. In the present study, we have tried to limit these influencing factors by only including retired subjects living in the same region (Guangzhou City) and by conducting the study over a short term (between June and November), which we thought would reduce the impact of work environments and weather. Finally, another limitation of the study includes the sample size. The number of patients enrolled in our study was limited, and further population-based studies should be done to confirm these results.

## 5. Conclusion

In this study, we used a visual function questionnaire concurrently with an objective physical activity assessment, and we demonstrated an adverse impact of glaucoma on psychological function and daily physical activity. We found adverse effects of glaucoma on social function, mental health, and daily activities. Moreover, patients with advanced-stage glaucoma showed limited physical activity. A correlation between low physical activity and deteriorated social function and mental health was found in patients with glaucoma. Further research is required to determine whether therapeutic strategies that increase physical activity might improve psychological function in these patients.

## Figures and Tables

**Table 1:** Clinical characteristics of the study subjects.

Characteristic	Glaucoma (no.)	Normal (no.)	*p* value
No. of patients	50	50	—
Age, *y* (mean [SD])	62.6 (5.3)	61.2 (4.3)	0.176
55–64	33	37	0.383
65–75	17	13	
Sex			
Male	20	26	0.229
Female	30	24	
Marital status			
Married	47	48	0.646
Divorced, separated, or single	3	2	
Education level			
Primary school	9	7	0.385
Junior middle school	16	17	
Senior middle school	11	18	
University	13	8	
Postgraduate	1	0	
Current smoker	5	5	1.000
Current drinking	6	4	0.507
Chronic diseases (mean [SD])	0.66 (0.79)	0.49 (0.68)	0.308
None	26	29	0.830
1-2	23	20	
>2	1	1	
Insurance	7	4	0.340
Glaucoma type			
POAG	19	—	—
PACG	31	—	
Early-stage glaucoma	9	—	
Moderate-stage glaucoma	8	—	
Advanced-stage glaucoma	33	—	
No. glaucoma eye drops used (mean [SD])	0.54 (0.96)	—	—
Season of baseline measure			
March–May	—	—	
June–August	32	25	0.157
September–November	18	25	
December–February	—	—	
BMI kg/m^2^ (mean [SD])	23.48 (2.57)	23.49 (3.56)	0.987
<25 kg/m^2^	33	35	0.668
>25 kg/m^2^	17	15	
Heart rate (mean [SD])	71.68 (12.07)	71.33 (9.12)	0.870
IOP (mmHg)			
Lower eye (mean [SD])	14.81 (3.85)	13.07 (2.20)	0.007
Higher eye (mean [SD])	18.04 (6.88)	13.91 (2.83)	<0.001
VA			
Better eye (mean [SD])	0.75 (0.18)	0.97 (0.17)	<0.001
Worse eye (mean [SD])	0.62 (0.23)	0.85 (0.20)	<0.001
VF			
MD better eye (mean [SD])	–8.00 (8.70)	–1.94 (2.59)	<0.001
MD worse eye (mean [SD])	–17.94 (10.16)	–3.33 (4.04)	<0.001

BMI = body mass index; IOP = intraocular pressure; VA = visual acuity; VF = visual field; SD = standard deviation.

**Table 2:** Differences in accelerometry data between patients with glaucoma and healthy control subjects.

Accelerometry data	Glaucoma	Normal	*p* value^*∗*^
Daily step count	11682.22 (2706.97)	12703.77 (2922.34)	0.102
MVPA (%)	13.15 (7.62)	14.24 (5.32)	0.422
Sleep quality (%)	91.04 (3.86)	90.51 (5.14)	0.570
Metabolic rate (%)	1.39 (0.13)	1.43 (0.24)	0.276

The data are presented as mean (SD).

**Table 3:** Differences in accelerometry data between patients with early-, moderate-, and advanced-stage glaucoma and healthy control subjects.

Accelerometry data	Diagnosis	Mean (SD)	Mean difference (SD)	*p* value^a^	*p* value^b^	*p* value^c^
Daily step count	Normal (ref)	12703.77 (2922.34)	—	—	—	—
	Early-stage glaucoma	13341.77 (2026.99)	638.00 (989.94)	0.521	—	—
	Moderate-stage glaucoma	12000.38 (2940.45)	–703.39 (1179.50)	0.553	0.352	-
	Advanced-stage glaucoma	11209.22 (2422.61)	–1494.55 (702.83)	0.037	0.051	0.530

MVPA (%)	Normal (ref)	14.24 (5.32)	—	—	—	—
	Early-stage glaucoma	11.87 (7.35)	–2.36 (2.38)	0.323	—	—
	Moderate-stage glaucoma	14.92 (13.39)	0.67 (2.65)	0.799	0.359	—
	Advanced-stage glaucoma	13.51 (5.94)	–0.73 (1.50)	0.628	0.509	0.607

Sleep quality (%)	Normal (ref)	90.51 (5.14)	—	—	—	—
	Early-stage glaucoma	91.59 (3.07)	1.07 (1.64)	0.515	—	—
	Moderate-stage glaucoma	93.98 (2.65)	3.47 (1.82)	0.061	0.293	—
	Advanced-stage glaucoma	90.26 (4.07)	–0.25 (1.03)	0.807	0.306	0.050

Metabolic rate (%)	Normal (ref)	1.43 (0.24)	—	—	—	—
	Early-stage glaucoma	1.43 (0.16)	0.00 (0.07)	0.965	—	—
	Moderate-stage glaucoma	1.32 (0.11)	–0.11 (0.07)	0.162	0.247	—
	Advanced-stage glaucoma	1.40 (0.11)	–0.03 (0.04)	0.454	0.619	0.342

The data are presented as mean (SD). *p* value^a^: early-, moderate-, and advanced-stage glaucoma vs. normal; *p* value^b^: moderate- and advanced-stage glaucoma vs. early-stage glaucoma; *p* value^c^: advanced-stage glaucoma vs moderate-stage glaucoma.

**Table 4:** Correlations between daily step counts and clinical variables in patients with glaucoma and healthy control subjects.

	Total		Glaucoma		Normal	
	Beta	*p* value	Beta	*p* value	Beta	*p* value
Age	–75.30	0.173	–182.21	0.025	68.27	0.414
Sex (female ref)	368.60	0.560	–543.71	0.545	1739.90	0.056
Marital status	2835.80	0.167	3009.86	0.279	2553.67	0.393
Education level	238.73	0.433	155.50	0.680	372.70	0.452
Chronic diseases	–868.90	0.145	–1406.47	0.111	–310.25	0.703
Insurance	–453.56	0.672	–42.59	0.974	–1622.30	0.358
Season of baseline measure	–142.00	0.813	1206.07	0.183	–1443.27	0.067
BMI kg/m^2^	–24.35	0.803	–88.89	0.588	2.67	0.983
VA						
Better eye	5685.85	<0.001	6981.01	0.002	5127.98	0.042
Worse eye	3995.29	0.002	5030.76	0.008	3620.20	0.091
VF						
MD better eye	93.17	0.064	114.35	0.024	–114.44	0.490
MD worse eye	65.95	0.030	104.28	0.007	–130.52	0.217
Social function	86.91	<0.001	78.303	<0.001	182.98	0.090
Mental health	63.39	0.001	53.32	0.019	86.74	0.034

## Data Availability

The data used to support the findings of this study are included within the article.
